# Neoadjuvant radiotherapy of early-stage breast cancer and long-term disease-free survival

**DOI:** 10.1186/s13058-017-0870-1

**Published:** 2017-06-30

**Authors:** Jan Poleszczuk, Kimberly Luddy, Lu Chen, Jae K. Lee, Louis B. Harrison, Brian J. Czerniecki, Hatem Soliman, Heiko Enderling

**Affiliations:** 1 Department of Integrated Mathematical Oncology, H. Lee Moffitt Cancer Center & Research Institute, 12902 Magnolia Drive, SRB 4, Tampa, FL 33612 USA; 2 Department of Cancer Imaging & Metabolism, H. Lee Moffitt Cancer Center & Research Institute, Tampa, FL 33612 USA; 3 Department of Biostatistics and Bioinformatics, H. Lee Moffitt Cancer Center & Research Institute, Tampa, FL 33612 USA; 4 Department of Radiation Oncology, H. Lee Moffitt Cancer Center & Research Institute, Tampa, FL 33612 USA; 5 Department of Breast Oncology, H. Lee Moffitt Cancer Center & Research Institute, Tampa, FL 33612 USA; 6 Department of Women’s Oncology and Experimental Therapeutics, H. Lee Moffitt Cancer Center & Research Institute, Tampa, FL 33612 USA; 7 Department for Mathematical Modeling of Physiological Processes, Nalecz Institute of Biocybernetics and Biomedical Engineering, Polish Academy of Sciences, Warsaw, Poland

**Keywords:** Postoperative radiotherapy, Preoperative radiotherapy, Early-stage breast cancer, Overall survival, Disease-free survival, Immune response

## Abstract

**Background:**

Compared with surgery alone, postoperative adjuvant radiotherapy (RT) improves relapse-free survival of patients with early-stage breast cancer. We evaluated the long-term overall and disease-free survival rates of neoadjuvant (presurgical) versus adjuvant RT in early-stage breast cancer patients.

**Methods:**

We used the Surveillance, Epidemiology, and End Results (SEER) database provided by the National Institutes of Health to derive an analytic dataset of 250,195 female patients with early-stage breast cancer who received RT before (*n* = 2554; 1.02%) or after (*n* = 247,641; 98.98%) surgery. Disease-free survival, defined as time to diagnosis of a second primary tumor at any location, was calculated from automated patient identification matching of all SEER records.

**Results:**

Partial and complete mastectomies were performed in 94.4% and 5.6% of patients, respectively. In the largest cohort of estrogen receptor-positive women who underwent partial mastectomy, the HR of developing a second primary tumor after neoadjuvant compared with adjuvant RT was 0.64 (95% CI 0.55–0.75; *P* < 0.0001). Overall survival was independent of radiation sequence (HR 1; *P* = 0.95). Neoadjuvant RT also resulted in a lower HR for second primary cancer among estrogen receptor-positive patients who underwent mastectomy compared with those who received adjuvant RT (HR 0.48, 95% CI 0.26–0.87; *P* = 0.0162).

**Conclusions:**

Neoadjuvant RT may significantly improve disease-free survival without reducing overall survival, especially for estrogen receptor-positive patients with early-stage breast cancer. This finding warrants further exploration of potential long-term benefits of neoadjuvant radiotherapy for early-stage breast cancer in a controlled, prospective clinical trial setting, with correlative studies done to identify potential mechanisms of superiority.

**Electronic supplementary material:**

The online version of this article (doi:10.1186/s13058-017-0870-1) contains supplementary material, which is available to authorized users.

## Background

Breast cancer is the most commonly diagnosed cancer type in women, accounting for around 41,000 deaths in the United States in 2015 [1]. Most patients with breast cancer are diagnosed at an early stage (61.1%) [1], largely as a result of widespread mammography screening programs. The standard of care for these patients is lumpectomy or mastectomy plus lymph node sampling followed by adjuvant radiotherapy (RT) to the tumor bed or the whole breast as indicated [2], which has been shown to reduce the risk of recurrence [3]. Postoperative RT to the chest wall and any part of the axillary bed at risk is also considered after total mastectomy in patients with stage T3 or T4 disease or those who are node-positive [2]. Neoadjuvant chemotherapy and RT before surgery are used in some locally advanced cases to debulk initially inoperable disease as an alternative to radical mastectomy [4–6]. Neoadjuvant accelerated partial breast irradiation can help reduce soft tissue toxicity because the clinical tumor target volume is smaller than the corresponding lumpectomy cavity [7, 8]. To date, no population-based analysis of the long-term effects of neoadjuvant RT has been performed. We designed a study to assess overall and cancer-free survival rates of patients with early-stage breast cancer who received either adjuvant or neoadjuvant RT.

## Methods

### Study design

We used the Surveillance, Epidemiology, and End Results (SEER) database (November 2013 submission), a National Cancer Institute (NCI)-initiated registry of cancer incidence and survival rates in the United States from 1972 to 2012, to derive a dataset of women with primary breast cancer treated with surgery and local breast RT. The database was accessed by using the SEER*Stat software provided by the NCI, as well as sets of ASCII files to restructure the data to calculate cancer-free survival of patients with a first primary breast tumor. Records were merged by patient identification number to identify dates of a second cancer diagnosis. Cancer-free survival, defined as time to diagnosis of a second primary tumor at any location, was calculated as the difference between the first and second diagnosis dates. Cancer-free survival was censored and set to overall survival for patients without any record of a second tumor diagnosis.

### Patients

**Fig. 1:**
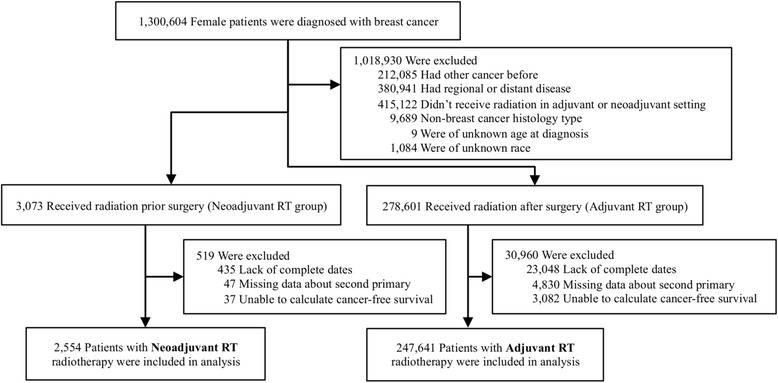
Study enrollment. Of 1,300,604 female breast cancer records present in the Surveillance, Epidemiology, and End Results database, 2554 patients who had radiotherapy (RT) before surgery (neoadjuvant RT) and 247,641 patients who had RT after surgery (adjuvant RT) were included in the analysis

All female patients diagnosed between 1973 and 2011 with carcinoma in situ or localized breast cancer (T1–T3) without prior cancer elsewhere were stratified into two groups: those who received localized breast radiation before surgery (neoadjuvant RT) and those who received localized breast radiation after surgery (adjuvant RT). Selected cases were lymph node-negative as per the SEER definition of localized and in situ disease. Patients with advanced-stage disease or insufficient demographic or treatment data were excluded from the study (Fig. 1). Baseline patient characteristics of both cohorts are presented in Table 1.

**Table 1:** Baseline characteristics of the stratified neoadjuvant and adjuvant radiotherapy cohorts

	Number of patients (%)				*P* value
	Neoadjuvant RT (*n* = 2554)		Adjuvant RT (*n* = 247,641)		
Age, years	58.6 ± 12.9		59.5 ± 12.2		<0.0001^a^
Year of diagnosis	1995.7 ± 7.2		2003.1 ± 6.5		<0.0001^a^
Race					
White	2348	(91.9%)	206,775	(83.5%)	<0.0001^b^
Black	127	(5%)	21,169	(8.5%)	<0.0001^b^
Other	79	(3.1%)	19,697	(8%)	<0.0001^b^
ER status					
Positive	1368	(53.6%)	168,797	(68.2%)	<0.0001^b^
Negative	353	(13.8%)	34,494	(13.9%)	0.88^b^
Unknown	833	(32.6%)	44,350	(17.9%)	<0.0001^b^
PR status					
Positive	1209	(47.3%)	144,228	(58.2%)	<0.0001^b^
Negative	492	(19.3%)	54,473	(22%)	0.0009^b^
Unknown	853	(33.4%)	48,940	(19.8%)	<0.0001^b^
Stage					
Carcinoma in situ	344	(13.5%)	52,691	(21.3%)	<0.0001^b^
T1	1390	(54.4%)	146,954	(59.3%)	<0.0001^b^
T2	294	(11.5%)	30,683	(12.4%)	0.1798^b^
T3	49	(1.9%)	3034	(1.2%)	0.0016^b^
Unknown	477	(18.7%)	14,279	(5.8%)	<0.0001^b^
Histology					
Ductal	2215	(86.7%)	213,143	(86.1%)	0.3397^b^
Lobular	146	(5.7%)	14,270	(5.8%)	0.9212^b^
Other	193	(7.6%)	20,228	(8.2%)	0.2614^b^
Type of surgery					
Partial mastectomy	2192	(85.8%)	233,946	(94.5%)	<0.0001^b^
Mastectomy	269	(10.5%)	10,632	(4.3%)	<0.0001^b^
Other	93	(3.6%)	3063	(1.2%)	<0.0001^b^

*ER* Estrogen receptor, *PR* progesterone receptor

Age and year of diagnosis are reported as mean ± SD

^a^ Wilcoxon rank-sum test with normal approximation

^b^ Pearson’s chi-square test

The medical reason for neoadjuvant RT is not recorded in the SEER database. In general, neoadjuvant RT for early-stage breast cancer would be prescribed to debulk tumors close to the skin or chest wall before surgery. Information about other systemic therapies is also not recorded; however, many of the patients with early-stage breast cancer likely received some form of endocrine therapy and/or chemotherapy [5].

### Statistical analyses

The Wilcoxon rank-sum test with normal approximation was used to compare continuous factors between neoadjuvant and adjuvant RT patient cohorts. Pearson’s chi-square test was used to compare categorical factors; each level of categorical factors was compared with all other levels combined between neoadjuvant and adjuvant patient cohorts. Median follow-up was estimated by Kaplan-Meier analysis with reversed meaning of status indicator [9].

Multivariate Cox proportional hazards models were used to estimate HRs [10]. Patients who were alive at the end of 20 years were censored for overall survival. Patients who did not have a record of a second tumor diagnosis within 20 years after their primary diagnosis were censored for cancer-free survival. Statistical analysis was performed using SAS version 9.4 software (SAS Institute, Cary, NC, USA) and R version 3.2.3 software (R Foundation for Statistical Computing, Vienna, Austria).

## Results

### Cancer-free survival

**Fig. 2:**
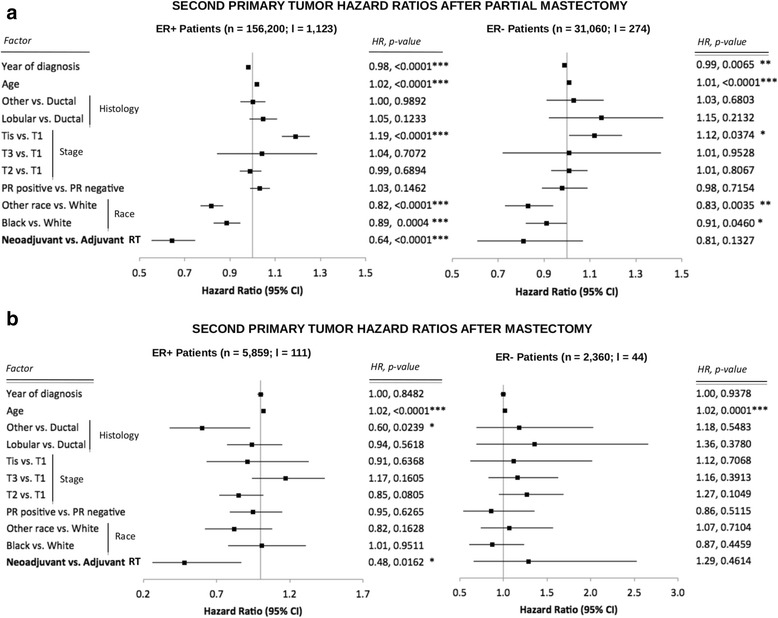
HRs of developing second primary tumors calculated from a multivariate Cox proportional hazards model. **a** Patients who underwent breast-conserving surgery. **b** Patients who underwent mastectomy. Shown are HRs, 95% CIs, and *P* values. *n* Total number of patients in each cohort, *I* Number of patients who received neodjuvant radiotherapy (RT) in each cohort, *ER* Estrogen receptor

**Fig. 3:**
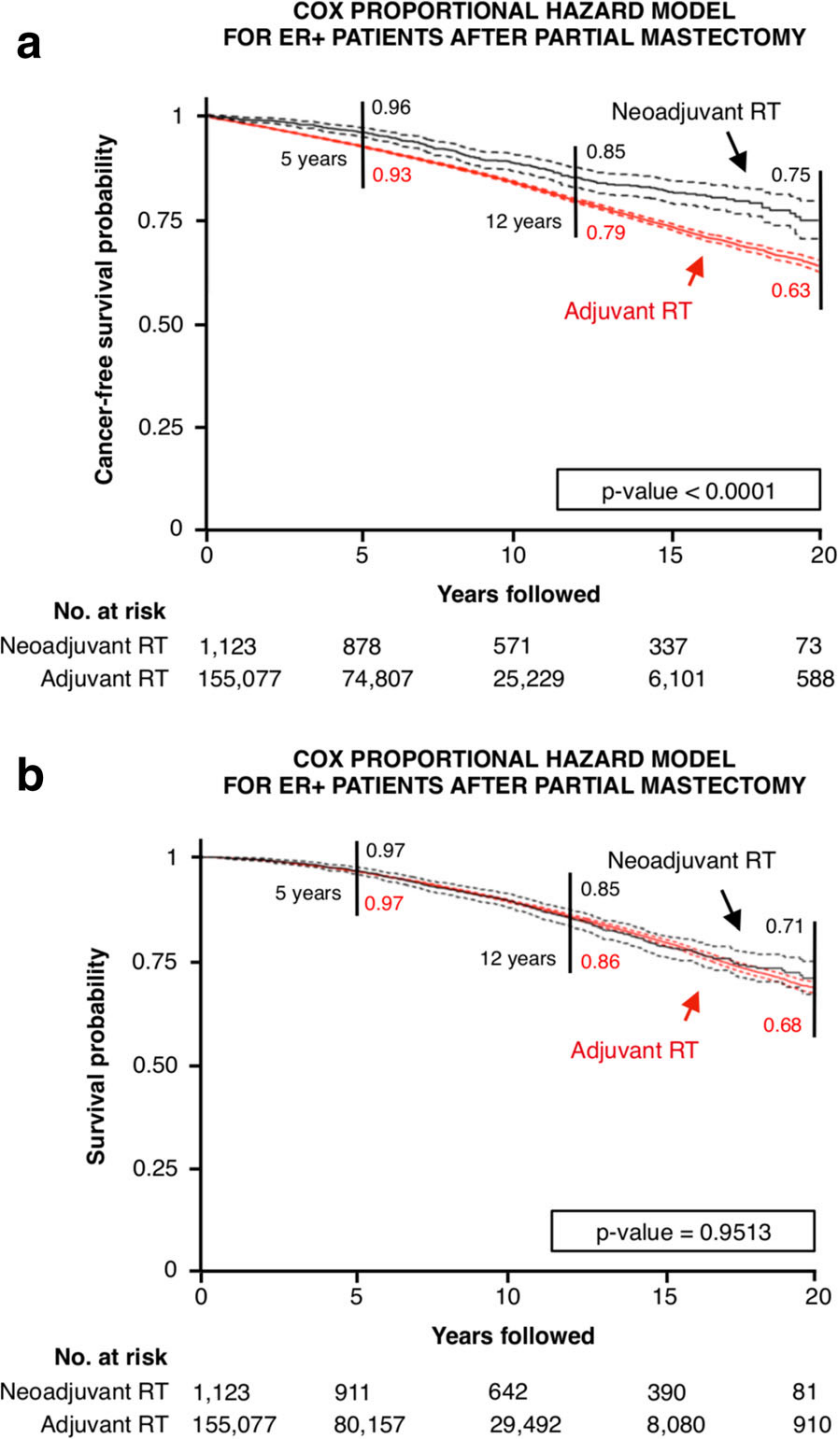
Survival curves for estrogen receptor-positive patients after partial mastectomy. Shown are (**a**) cancer-free and (**b**) overall survival curves with 95% CIs. *RT* radiotherapy

Adjuvant and neoadjuvant RT cohorts had significantly different distributions of potentially confounding factors, including years of diagnosis, hormone receptor status, tumor stage, and surgical procedure (Table 1). For the multivariate Cox proportional hazards model, estrogen receptor (ER) status (positive or negative) and surgery type (partial mastectomy or mastectomy) did not fulfill the assumption of proportionality and were stratified into separate cohorts for analyses (Fig. 2). Independent of surgery type, the HR for second primary cancer diagnosis among the ER-positive patients after neoadjuvant RT compared with adjuvant RT was significantly lower (HR 0.64, *P* < 0.0001; and HR 0.48, *P* = 0.0162 for partial mastectomy and mastectomy, respectively) (Fig. 2). Patients who underwent partial mastectomy had a slightly lower HR of second primary tumor diagnosis if treated more recently (HR for year of diagnosis ≤0.99, *P* < 0.01). Interestingly, patients staged with carcinoma in situ who underwent breast-conserving surgery had significantly higher incidences of second primary tumors compared with patients with stage T1 tumors (HR 1.19, *P* < 0.0001; and HR = 1.12, *P* = 0.0374 for ER-positive and ER-negative patients, respectively) (Fig. 2). Cancer-free survival probability of the largest cohort of ER-positive patients treated with partial mastectomy (*n* = 155,077; 62% of the total analyzed population) was 12% higher after neoadjuvant RT than after adjuvant RT (0.75 vs. 0.63; *P* < 0.0001) (Fig. 3a).

### Mortality

Median follow-up durations for the adjuvant and neoadjuvant RT cohorts were 7 years and 16.3 years, respectively, with maximums of 38.9 years and 38.6 years. Data showed that 36,607 patients (14.8%) with adjuvant RT treatment had the vital status recode “dead” at the end of follow-up versus 896 patients (35.1%) with neoadjuvant RT treatment.

**Fig. 4:**
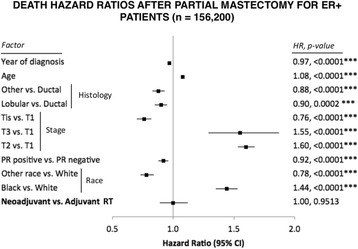
HRs of death calculated from a multivariate Cox proportional hazards model for patients diagnosed with estrogen-positive tumors who underwent breast-conserving surgery. Shown are HRs, 95% CIs, and *P* values. *PR* Progesterone receptor

The proportionality assumption required for multivariate Cox proportional hazards analysis was violated for most factors in the mastectomy and ER-negative partial mastectomy cohorts, requiring further stratification. Univariate analyses, however, resulted in insufficient patient numbers in the neoadjuvant RT groups, making the comparison with adjuvant RT infeasible. Multivariate Cox proportional hazards analysis assumptions were satisfied for all variables in the largest cohort of patients with ER-positive tumors who underwent breast-conserving surgery. Interestingly, there was no mortality hazard of neoadjuvant RT compared with standard-of-care adjuvant RT (HR 1.00, *P* = 0.9513), with survival curves being indistinguishable at 20-year follow-up (Fig. 3b). All other factors, including age, race, and tumor stage, however, significantly affected mortality (Fig. 4). Patients diagnosed with T2 and T3 tumors had the highest increases in mortality risk (HR 1.55 and HR 1.60, respectively; *P* < 0.0001) versus patients with T1 tumors.

## Discussion

Although adjuvant RT has significantly improved patient prognosis after breast-conserving surgery for early-stage breast cancer [3], only 63% of patients remain cancer-free within 20 years of primary treatment. RT enhances tumor-specific immune responses in well-established tumors [11–17]. Recent studies have demonstrated that radiation of the bulk tumor can activate robust antitumor immune responses [18, 19], potentially converting the tumor into a patient-specific in situ vaccine capable of re-educating the immune system to recognize and reject cancer [20, 21]. This synergy stems from the fact that radiation induces cell stress and immunogenic cell death, thereby exposing a wealth of previously hidden tumor-associated antigens, stress proteins, and danger-associated molecular patterns, which are endogenous immune adjuvants [22]. Thus, it is conceivable that neoadjuvant RT applied to the large bulk of disease activates a robust antitumor immunity, which is absent after postoperative RT to the tumor bed. Radiation-induced antitumor immunity may help eradicate subclinical disease in the ipsilateral and contralateral breast as well as distant micrometastases, possibly leading to an immune memory that vaccinates against future tumors [20]. This hypothesis motivated the analysis of long-term outcomes of neoadjuvant RT compared with standard-of-care adjuvant RT in patients with early-stage breast cancer. The requirement of assumed proportionality for the multivariate Cox proportional hazards model was violated for the potentially confounding factors of ER status (positive or negative) and surgical procedure (partial or complete mastectomy). Delayed recurrences occur primarily in ER-positive early-stage breast cancers [23], thereby changing the proportionality of recurrence curves before and after 5-year follow-up.

Our results suggest that RT before surgery reduces the incidence of second primary tumors without decreasing overall survival rates. A reduced second cancer risk attributable to radiation-induced antitumor immunity could explain the significantly higher incidences of second primary tumors in patients with carcinoma in situ but not in patients with T2 or T3 tumors compared with patients with stage T1 tumors treated with partial mastectomy. Carcinoma in situ cancer cells are unlikely to have strayed beyond the lumpectomy margin to become exposed to radiation and induce a robust immune response against circulating tumor cells or microscopic reservoirs elsewhere. Of note is that black women are significantly less likely to be diagnosed with second primary tumors; however, black women have a much higher mortality hazard. This disparity may indicate a severe lack of long-term follow-up and second cancer diagnoses in black women [24].

The number of ER-negative patients in the analyzed dataset is insufficient to draw statistically significant conclusions about second primary cancer incidence (Table 1 and Fig. 2). For the largest cohort of ER-positive patients who underwent partial mastectomy, data analysis showed that neoadjuvant radiation significantly improved cancer-free but not overall survival. To explain that unintuitive result, we calculated relative survival (ratio of observed survival to expected survival) statistics for both neoadjuvant and adjuvant RT cohorts, which are indistinguishable from the general population, regardless of RT sequencing (Additional file 1: Figure S1). Relative survival curves indicate that patients with early-stage breast cancer are very well treated with a very small incidence of life-threatening relapses. Thus, even if there was a vaccination-type response against distant metastases or secondary tumors, such a response would not be detectable in overall survival, even for large cohorts of patients. The highly skewed nature of the dataset, with 99% of patients having received adjuvant RT vs. 1% neoadjuvant RT, and the large difference in median follow-up for adjuvant (7 years) vs. neoadjuvant (16.3 years) RT cohorts are potential biases for the statistical analysis. However, bootstrap analyses cohorts matched for size and year of diagnosis confirm the presented results of the entire patient cohort (Additional file 1: Tables S1–S4). Moreover, cancer-free and overall survival results were additionally confirmed in propensity-matched cohorts (Additional file 1: Figure S3).

Older patients in all stratified groups consistently had a slightly higher risk of developing second primary tumors (HR ≥1.01, *P* = 0.0001). The slightly higher risk for older patients of developing secondary primary tumors was confirmed in the bootstrap analysis of patient cohorts matched for size and year of diagnosis (Additional file 1: Tables S1–S4). The HR of 1.01 may be statistically significant owing to the large sample size, but it may not allow biological or clinical interpretation. We could speculate that older patients may have received different therapies, including lower total radiation doses and less additional systemic therapy.

The limitation of the presented analysis is its retrospective nature with all applicable biases, absent systemic treatment data, and no information regarding selection criteria for neoadjuvant RT recorded in the SEER database. Long-term cancer-free survival after neoadjuvant RT needs to be validated in prospective, randomized clinical trials with control for confounding factors, including radiation protocols (total dose, fractionation) and concurrent systemic therapies.

A complete course of neoadjuvant RT may introduce concerns for subsequent lumpectomy, including skin burn and fibrosis. This could be prevented through neoadjuvant accelerated partial breast irradiation or stereotactic protocols [23]. The observed benefit of neoadjuvant RT aligns with the growing body of literature of the immune activation effects of radiation, including shrinking of untreated metastases outside the radiation field [25–27]. To fully harness the synergy of radiation and the immune system, it may be sufficient to deliver limited immune-priming radiation (35 Gy in ten fractions over 2 weeks [28], 24 Gy in three fractions [29]) before surgery and shortened courses of adjuvant standard fractionation protocols. Many ER-positive patients with early-stage breast cancer are currently treated with lumpectomy, adjuvant radiation, and endocrine therapy alone. One could envision a short course of preoperative partial breast RT for a clinical early-stage, ER-positive, HER2-negative tumor, followed by a period of preoperative endocrine therapy, to allow time for the immune response to form; then the patient could undergo lumpectomy and lymph node sampling. If patients remain lymph node-negative and are suitable for continued endocrine therapy alone, then this sequence may reduce additional delayed recurrences without significant extra toxicity. Although this group is perceived as having an excellent prognosis overall, the cumulative incidence of long-term recurrences seen in the ER-positive SEER cohort would argue that the simple switch from adjuvant to neoadjuvant RT may result in a meaningful benefit for a large number of women over time. For higher-stage or more aggressive disease, neoadjuvant radiation-induced immunity may require enhancement by subsequent chemoimmunotherapy with agents such as weekly taxanes plus an anti-CTLA4 antibody [26] or anti-programmed death 1 antibody [30].

## Conclusions

To our knowledge, this is the first study to explore the long-term incidence of second primary cancers and survival for patients with early-stage breast cancer who received RT adjuvant or neoadjuvant to surgery. Our findings show that the hazard of developing second primary cancer is significantly lower when RT is applied before surgery than after surgery, especially for ER-positive patients. Neoadjuvant radiation does not decrease overall survival for patients with early-stage breast cancer. These findings are worthy of a prospective clinical trial to improve local control while decreasing the risk of distant metastases and to identify the potential contribution of neoadjuvant vs. adjuvant radiation-induced immunity.

## Additional file

Additional file 1: Relative survival and bootstrap analyses. Relative survival for ER-positive patients who underwent partial mastectomy (stratified by radiation sequencing) and bootstrap analyses for neoadjuvant and adjuvant RT cohorts matched for size and year of diagnosis. (DOCX 749 kb)

## Abbreviations

ER: Estrogen receptor
NCI: National Cancer Institute
PR: Progesterone receptor
RT: Radiotherapy
SEER: Surveillance, Epidemiology, and End Results database
