# Supplementary material for: Neoadjuvant radiotherapy of early-stage breast cancer and long-term disease-free survival
Source: Breast Cancer Res. 2017 Jun 30;19:75. doi: 10.1186/s13058-017-0870-1 (PMC5493088; doi:10.1186/s13058-017-0870-1)
Supplement: Additional file 1: — Relative survival and bootstrap analyses. Relative survival for ER-positive patients who underwent partial mastectomy (stratified by radiation sequencing) and bootstrap analyses for neoadjuvant and adjuvant RT cohorts matched for size and year of diagnosis. (DOCX 749 kb) [file 13058_2017_870_MOESM1_ESM.docx]

**Supplementary materials**

**„Neoadjuvant Radiotherapy of Early-Stage Breast Cancer and Long-Term Disease-Free Survival”**

Jan T. Poleszczuk, Kimberly Luddy, Lu Chen, Jae K. Lee, Louis B. Harrison, Brian J Czerniecki, Hatem Soliman and Heiko Enderling

1. **Relative survival of ER positive patients after partial mastectomy**

To explain the difference in cancer-free but not overall survival between adjuvant and neoadjuvant radiotherapy cohorts of ER positive patients after partial mastectomy (compare Fig. 3 in the manuscript) we calculated relative survival statistics for both cohorts. Age-, race- and gender-matched expected survival for each group was calculated using the Ederer II method using expected survival life tables provided by SEER and distributed with the SEER*Stat software. Relative survival (ratio of observed survival to expected survival) was adjusted if exceeding 100% and in case of increasing values (correction with the value from previous period). Standard error for observed survival was obtained using the Greenwood formula and statistical significance between each relative survival curves at specific time point was calculated using Z-test.

The relative survival up to 20 years of ER positive patients after partial mastectomy is almost indistinguishable from the general population regardless of radiotherapy sequencing (at 20 years point relative survival = 97% adjuvant RT vs. 96% neoadjuvant RT; p>0.49, **Fig. S1**).


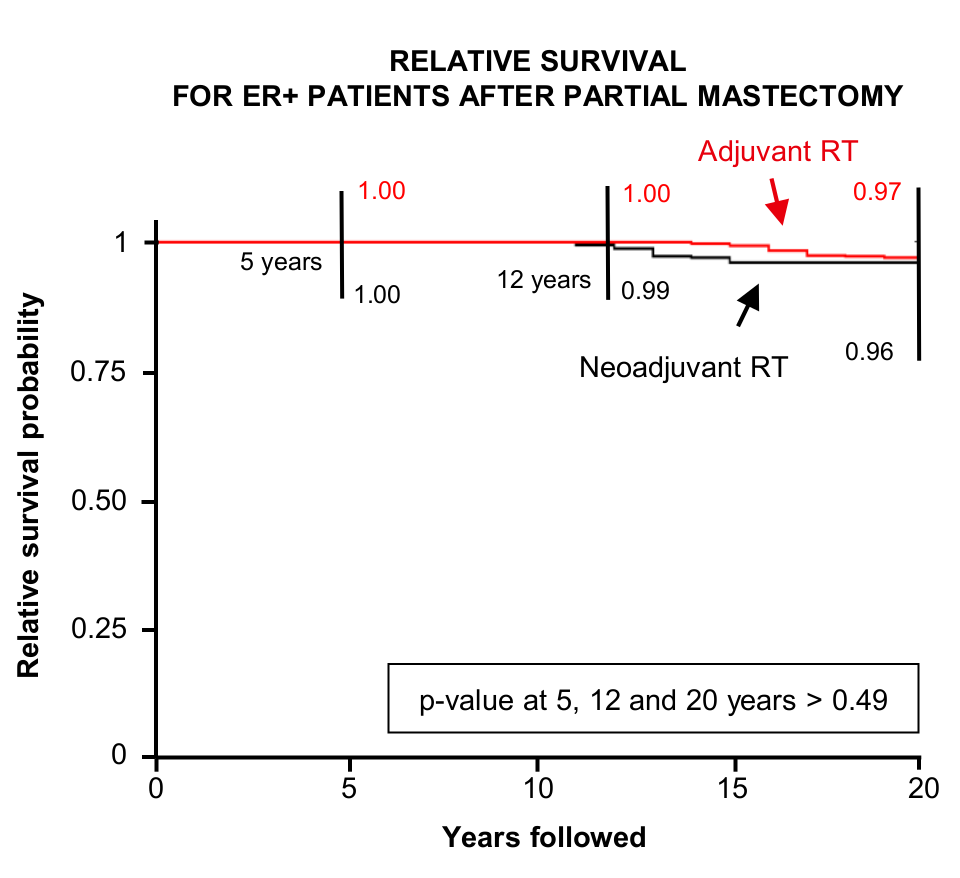


**Figure S1.** Relative survival curves for estrogen receptor positive (ER+) patients that underwent partial mastectomy before (Adjuvant RT) or after radiotherapy (Neoadjuvant RT).

**3. Bootstrap analysis for size and year-of-diagnosis matched cohorts**

Structure of the data used for the analysis has two potential biases: 1) the highly skewed nature of the data set with 99% of patients having received adjuvant RT (vs. 1% neoadjuvant RT); 2) large difference in median follow-up for adjuvant (7 years) vs. neoadjuvant (16.3) RT cohorts. The difference in median follow-up is the result of large difference in the distribution of cases with respect to the year of diagnosis between both cohorts, with many more cases diagnosed after 2000 in the adjuvant RT group (**Fig. S2**).

To systematically address both potential biases we bootstrapped the data. For each year of diagnosis we calculated the number of neoadjuvant RT cases, and selected (*i*) the same number of patients and (*ii*) 10 times the number of patients at random from the year-matched adjuvant cohort. Because of the random nature of this bootstrap we repeated the analysis for 5 random cohorts each. Multivariate Cox analysis for cancer-free and observed survival was performed for each randomly generated data set (unchanged neoadjuvant RT part + bootstrapped adjuvant RT cohort) as was done for the complete data set.

The median follow-up for year-of-diagnosis matched neoadjuvant RT cohort was 16.3 years and between 15.9 and 16 years for all adjuvant RT cohorts. Multivariate Cox analysis for each of the 10 randomly generated datasets consistently confirmed the analysis results of the total cohorts as originally reported (**Tables S1 – S4**). The hazard ratio of secondary cancer incidence for the largest cohort of ER+ patients who underwent partial mastectomy (HR=0.64) was confirmed to be statistically significant in all cohorts (adjuvant RT size matched (average HR=0.64 +/- 0.02; **Table S1**); adjuvant RT with 10 times more patients (HR=0.63 +/- 0.006; **Table S3**)).

**
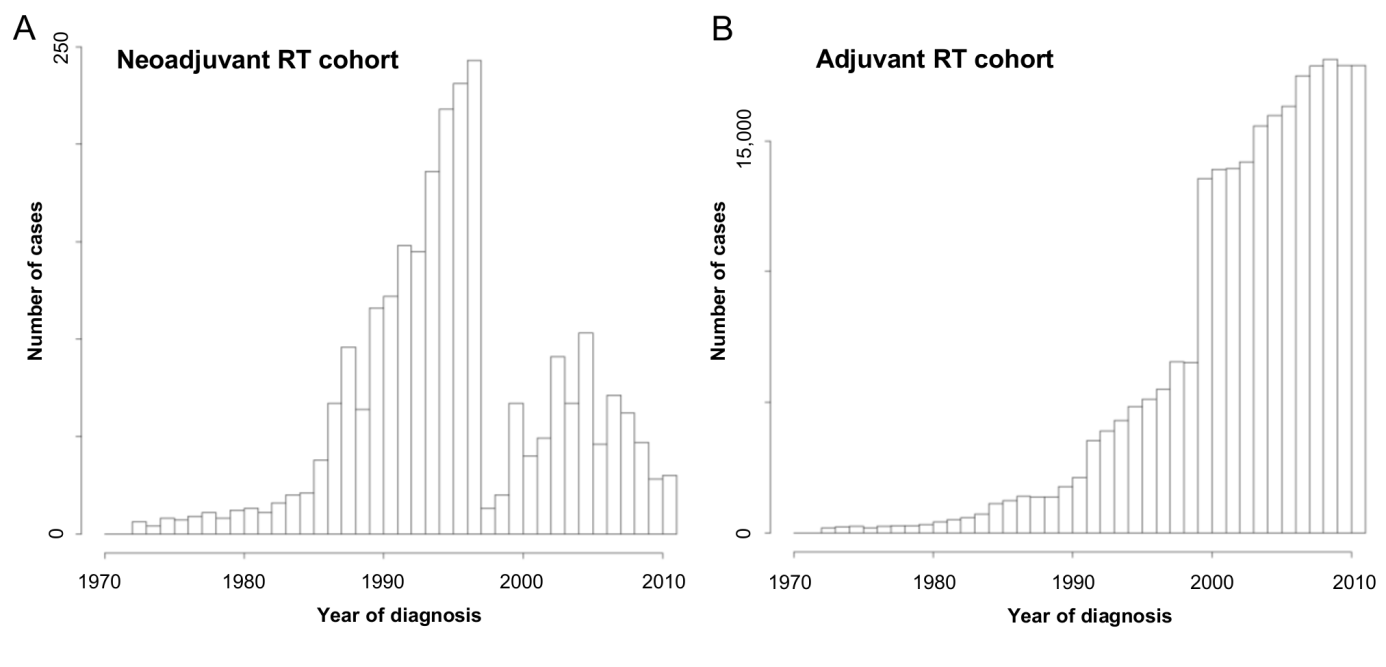
**

**Figure S2.** Year of diagnosis distribution for neoadjuvant (A) and adjuvant RT (B) cohorts. Shown are number of cases per year.

**2. Propensity matching**

To confirm the statistical analysis results obtained by the multivariate Cox model we performed cancer-free and overall survival analyses on propensity matched neoadjuvant and adjuvant radiotherapy (RT) cohorts. The propensity score matched analysis is emulating cohorts with comparable distributions of all potentially confounding factors (**Table S0**), which was achieved by using the *matchit* function from the MatchIt package for R software.

**Table S0.** Baseline characteristics of the propensity matched cohorts.

|  | Neoadjuvant RT | Adjuvant RT | p-value |
| --- | --- | --- | --- |
| N | 1,552 | 1,552 |  |
| Age (mean (sd)) | 58.33 (12.69) | 58.69 (12.62) | 0.733 |
| Year of diagnosis (mean (sd)) | 1998.35 (5.88) | 1998.23 (5.85) | 0.573 |
| Race (%) |  |  | 0.685 |
| White | 1,419 (91.4) | 1,406 (90.6) |  |
| Black | 78 (5) | 83 (5.3) |  |
| Other | 55 (3.5) | 63 (4.1) |  |
| ER positive (%) | 1,234 (79.5) | 1,242 (80) | 0.754 |
| PR positive (%) | 1,106 (71.3) | 1,123 (72.4) | 0.523 |
| Stage (%) |  |  | 0.649 |
| T1 | 1,123 (72.4) | 1,153 (74.3) |  |
| T2 | 252 (16.2) | 236 (15.2) |  |
| T3 | 40 (2.6) | 34 (2.2) |  |
| Tis | 137 (8.8) | 129 (8.3) |  |
| Histology (%) |  |  | 0.648 |
| Ductal | 1,340 (86.3) | 1,356 (87.4) |  |
| Lobular | 97 (6.2) | 86 (5.5) |  |
| Other | 115 (7.4) | 110 (7.1) |  |
| Mastectomy (%) | 155 (10) | 128 (8.2) | 0.105 |

Survival analyses on the matched cohorts using Kaplan-Meier estimators confirm that patients treated with neoadjuvant RT have significantly lower incidences of secondary cancers (cancer-free survival after 20 years 0.68 vs. 0.55; p-value=1.02e-06) and the same 20-years overall survival as the adjuvant RT cohort (0.59 vs. 0.54; **Fig. S3**). These results are in strong agreement with the multivariate Cox analysis (c.f. Fig. 3).

**
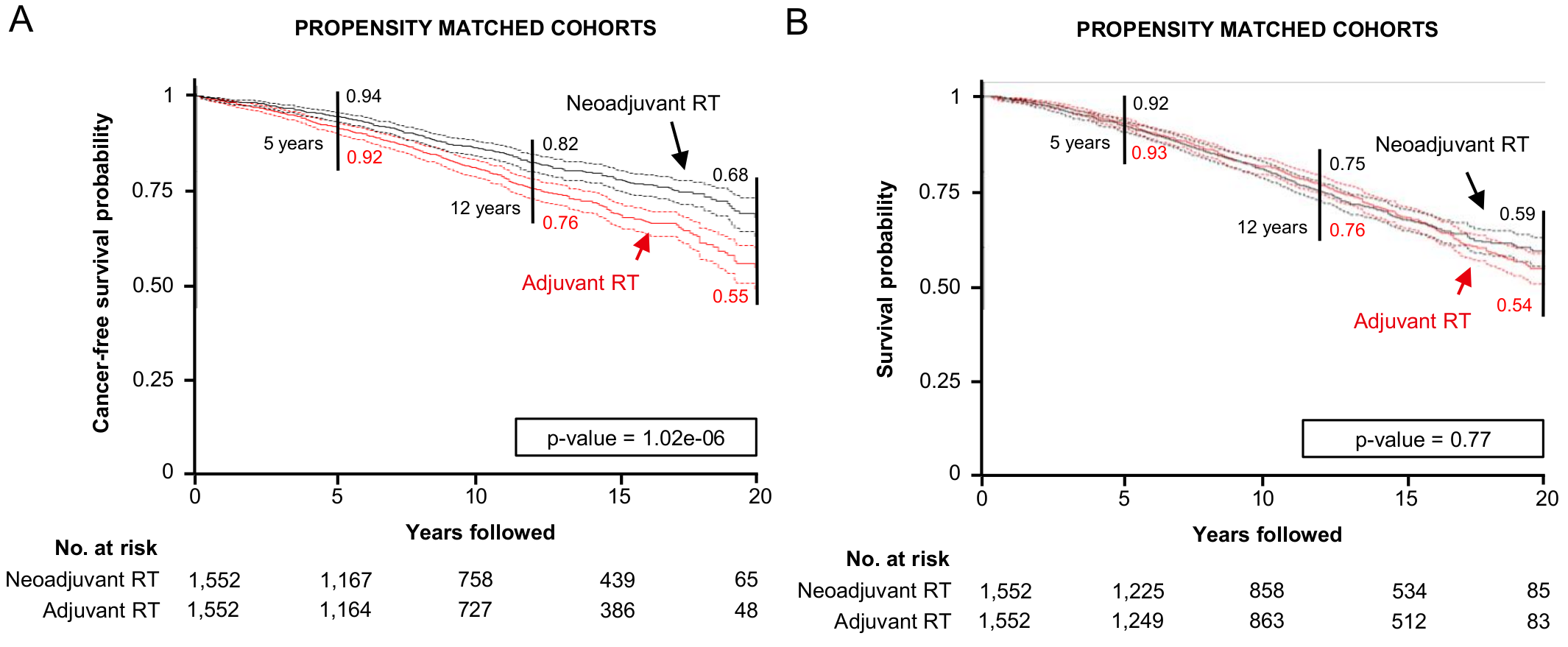
**

**Figure S3.** Kaplan-Maier cancer-free (A) and overall (B) survival curves estimated for propensity matched adjuvant and neoadjuvant radiotherapy (RT) cohorts. p-values are calculated with the log-rank test.

**Table S1.** Results of the multivariate Cox analysis of cancer-free survival for 5 randomly generated datasets consisted of original unchanged neoadjuvant radiation cohort and randomly sampled year-of-diagnosis matched adjuvant RT cohort with the same number of patients as the neoadjuvant RT group. Shown are hazard ratios.

|  | ER positive patients after partial mastectomy | | | | | ER positive patients after mastectomy | | | | | ER negative patients after partial mastectomy | | | | | ER negative patients after mastectomy | | | | |
| --- | --- | --- | --- | --- | --- | --- | --- | --- | --- | --- | --- | --- | --- | --- | --- | --- | --- | --- | --- | --- |
| Batch | #1 | #2 | #3 | #4 | #5 | #1 | #2 | #3 | #4 | #5 | #1 | #2 | #3 | #4 | #5 | #1 | #2 | #3 | #4 | #5 |
| HR |  | | | | |  | | | | |  | | | | |  | | | | |
| *Year of diagnosis* | 0.98 | 0.99 | 0.99 | 0.99 | 0.98 | 0.97 | 0.97 | 1.07 | 0.97 | 1.08 | 0.99 | 0.99 | 0.99 | 1.02 | 0.99 | 0.86 | 1.05 | 1.02 | 1.00 | 0.95 |
| *Age* | **1.02*** | **1.02*** | **1.01*** | **1.01*** | **1.01** | 1.04 | 1.00 | **1.05*** | 1.02 | 1.02 | **1.02*** | **1.02*** | **1.02*** | 1.00 | **1.02*** | 1.05 | **1.11*** | 1.04 | 1.04 | 1.06 |
| *Other vs. Ductal* | 1.32 | 1.36 | 1.17 | **1.43*** | **1.40*** |  |  |  |  |  | 1.16 | 0.95 | 0.96 | 0.54 | 0.92 | **14.19*** | **46.60*** | 11.01 | 7.84 | **21.15*** |
| *Lobular vs. Ductal* | 1.16 | **1.75*** | **1.42*** | **1.46*** | 1.26 | 1.32 | 0.74 | 0.30 | 0.76 | 0.35 | 0.99 | 0.69 | 1.75 | 0.83 | 1.89 | 9.13 | 6.47 | 8.30 | 8.74 | 7.56 |
| *Tis vs. T1* | **1.73*** | 1.32 | 1.42 | **1.72*** | **1.67*** | 3.68 | 4.04 | 3.10 | **14.72*** | 1.15 | **1.91*** | 1.15 | **1.92*** | 1.52 | **2.21*** | 3.91 | 3.55 | 1.43 | 3.61 | 3.87 |
| *T3 vs. T1* | 1.54 | 0.82 | 0.68 | 0.79 | 0.50 | **6.27*** | 2.23 | **5.32*** | 2.39 | 2.72 |  |  |  |  |  | 0.43 |  | 2.22 | 1.75 | 4.13 |
| *T2 vs. T1* | **1.38*** | 1.00 | 1.05 | 0.94 | 1.07 | 1.19 | 0.94 | 0.82 | 0.79 | 0.65 | 1.33 | 1.18 | 1.02 | 0.89 | **1.55*** | 1.46 | 0.77 | 2.00 | 2.70 | 1.53 |
| *PR+ vs. PR-* | 0.90 | 1.17 | **1.44*** | **1.78*** | 1.30 | 2.11 | 3.10 | **10.54*** | 0.68 | 0.70 | 0.86 | 1.26 | 0.85 | 1.21 | 0.84 | 0.00 | 5.38 |  |  |  |
| *Other race vs. White* | 0.65 | 0.67 | 0.68 | 0.75 | 0.79 | 0.00 | 0.98 |  | 0.88 | 1.06 | **0.23*** | 1.02 | 0.63 | 0.32 | 0.59 | 2.06 |  |  |  |  |
| *Black vs. White* | 0.82 | 0.85 | 1.01 | 0.96 | 0.81 | 0.75 | 0.69 | 1.10 | 1.56 | 2.23 | 0.68 | 0.69 | 0.87 | 0.90 | 0.51 | 0.13 |  |  |  |  |
| *Neoadjuvant vs Adjuvant RT* | **0.63*** | **0.66*** | **0.65*** | **0.64*** | **0.62*** | **0.24*** | 0.55 | 0.40 | **0.22*** | 0.51 | 0.70 | 0.93 | 0.74 | 0.85 | **0.63*** | 1.77 | 1.43 | 0.83 | 0.80 | 1.16 |

* p-value < 0.05

**Table S2.** Results of the multivariate Cox analysis of overall survival for 5 randomly generated datasets consisted of original unchanged neoadjuvant radiation cohort and randomly sampled year-of-diagnosis matched adjuvant RT cohort with the same number of patients as the neoadjuvant RT group. Shown are hazard ratios only for estrogen positive patients after partial mastectomy as in the main text.

|  | ER positive patients after partial mastectomy | | | | |
| --- | --- | --- | --- | --- | --- |
| Batch | #1 | #2 | #3 | #4 | #5 |
| HR |  | | | | |
| *Year of diagnosis* | **0.97*** | 0.99 | 0.98 | **0.97*** | **0.97*** |
| *Age* | **1.08*** | **1.07*** | **1.08*** | **1.08*** | **1.08*** |
| *Other vs. Ductal* | 0.88 | 0.80 | 0.76 | 0.72 | 0.80 |
| *Lobular vs. Ductal* | 1.02 | 1.24 | 0.91 | 1.03 | 0.95 |
| *Tis vs. T1* | 0.86 | 0.83 | 0.75 | 0.79 | 1.13 |
| *T3 vs. T1* | 1.43 | 1.70 | 1.49 | 1.45 | 1.74 |
| *T2 vs. T1* | **1.83*** | **1.41*** | **1.65*** | **1.73*** | **1.74*** |
| *PR+ vs. PR-* | 0.80 | 1.01 | 1.01 | 0.90 | 0.97 |
| *Other race vs. White* | 0.87 | 0.80 | 0.85 | 0.89 | 0.73 |
| *Black vs. White* | 1.34 | 1.36 | 1.21 | 0.98 | 1.02 |
| *Neoadjuvant vs Adjuvant RT* | 1.09 | 1.00 | 0.97 | 1.06 | 0.95 |

* p-value < 0.05

**Table S3.** Results of the multivariate Cox analysis of cancer-free survival for 5 randomly generated datasets consisted of original unchanged neoadjuvant radiation cohort and randomly sampled year-of-diagnosis matched adjuvant RT cohort with 10 times more patients than the neoadjuvant RT group. Shown are hazard ratios.

|  | ER positive patients after partial mastectomy | | | | | ER positive patients after mastectomy | | | | | ER negative patients after partial mastectomy | | | | | ER negative patients after mastectomy | | | | |
| --- | --- | --- | --- | --- | --- | --- | --- | --- | --- | --- | --- | --- | --- | --- | --- | --- | --- | --- | --- | --- |
| Batch | #1 | #2 | #3 | #4 | #5 | #1 | #2 | #3 | #4 | #5 | #1 | #2 | #3 | #4 | #5 | #1 | #2 | #3 | #4 | #5 |
| HR |  | | | | |  | | | | |  | | | | |  | | | | |
| *Year of diagnosis* | **0.99*** | **0.99*** | **0.99*** | **0.98*** | **0.98*** | 1.02 | 1.02 | 1.02 | 1.02 | 0.99 | 1.00 | 1.01 | 1.00 | 1.01 | 0.98 | 0.98 | 1.01 | 0.96 | 0.98 | 0.92 |
| *Age* | **1.02*** | **1.02*** | **1.02*** | **1.01*** | **1.02*** | **1.02*** | **1.03*** | **1.02*** | **1.02*** | **1.03*** | **1.01*** | **1.01*** | **1.02*** | **1.01*** | **1.01*** | 1.01 | **1.02*** | 1.01 | 1.01 | 1.01 |
| *Other vs. Ductal* | 0.99 | 1.02 | 0.95 | 1.02 | 1.03 | 0.21 | 0.14 |  | 0.60 | 0.17 | 0.93 | 0.97 | 1.15 | 1.02 | 0.77 | 1.14 | 0.94 | 2.67 | 1.93 | 2.45 |
| *Lobular vs. Ductal* | 0.99 | 1.14 | 1.01 | **1.17*** | 1.06 | 1.24 | 0.95 | 1.11 | 1.10 | 0.90 | 1.14 | 1.16 | 1.23 | 1.37 | 1.40 | 7.01 | 2.09 | **4.75*** | 0.73 | 3.52 |
| *Tis vs. T1* | 1.06 | **1.33*** | **1.31*** | 1.18 | **1.40*** | 2.07 | 1.88 | 1.72 | 0.66 | 1.96 | 1.23 | **1.46*** | 1.26 | 1.19 | 1.17 | 1.83 | 1.66 | 1.71 | 1.27 | 1.11 |
| *T3 vs. T1* | 1.57 | 1.77 | 1.30 | 1.10 | 0.96 | 1.09 | **2.14*** | 1.16 | 1.10 | 1.66 | 1.48 | 0.43 | 0.17 | 0.75 | 0.38 | 0.47 | 1.27 | 0.86 | 1.24 | 0.97 |
| *T2 vs. T1* | 0.99 | 1.03 | 1.00 | 0.95 | 1.10 | 0.85 | 0.96 | 0.85 | 0.72 | 0.97 | 1.09 | 1.13 | 1.11 | 1.17 | 1.06 | **2.15*** | 1.26 | **2.35*** | **2.04*** | 1.74 |
| *PR+ vs. PR-* | 1.07 | 0.99 | 1.06 | 1.06 | 1.08 | 1.46 | 1.30 | 0.73 | 1.07 | 1.05 | 0.98 | 1.11 | 1.02 | 1.10 | 1.09 | 0.28 | 0.89 | 0.45 | 0.87 | 1.10 |
| *Other race vs. White* | 0.88 | **0.77*** | **0.81*** | **0.77*** | **0.73*** | 1.46 | 0.96 | 1.38 | 0.98 | 1.12 | 0.82 | **0.73*** | 0.86 | 0.75 | 0.80 | **2.74*** | 1.55 | 2.01 | 0.74 | 2.16 |
| *Black vs. White* | 0.91 | **0.78** | **0.83** | 0.85 | **0.80** | 0.95 | 1.39 | 1.12 | 0.95 | 1.01 | 0.87 | 1.00 | 0.93 | **0.75*** | 1.00 | 1.89 | 1.34 | 1.07 | 0.62 | 0.63 |
| *Neoadjuvant vs Adjuvant RT* | **0.63*** | **0.62*** | **0.63*** | **0.64*** | **0.62*** | **0.51*** | **0.50*** | **0.52*** | **0.38*** | **0.49*** | 0.79 | 0.81 | 0.81 | 0.80 | 0.86 | 1.16 | 1.06 | 1.62 | 1.47 | 1.66 |

* p-value < 0.05

**Table S4.** Results of the multivariate Cox analysis of overall survival for 5 randomly generated datasets consisted of original unchanged neoadjuvant radiation cohort and randomly sampled year-of-diagnosis matched adjuvant RT cohort with 10 times more patients than the neoadjuvant RT group. Shown are hazard ratios only for estrogen positive patients after partial mastectomy as in the main text.

|  | ER positive patients after partial mastectomy | | | | |
| --- | --- | --- | --- | --- | --- |
| Batch | #1 | #2 | #3 | #4 | #5 |
| HR |  | | | | |
| *Year of diagnosis* | **0.97*** | **0.97*** | **0.97*** | **0.98*** | **0.97*** |
| *Age* | **1.08*** | **1.08*** | **1.08*** | **1.08*** | **1.08*** |
| *Other vs. Ductal* | 0.95 | **0.87*** | **0.86*** | 0.89 | **0.83*** |
| *Lobular vs. Ductal* | 0.95 | 0.97 | 1.06 | 1.02 | 1.01 |
| *Tis vs. T1* | **0.75*** | **0.78*** | **0.81*** | **0.80*** | **0.78*** |
| *T3 vs. T1* | **2.01*** | **1.86*** | **1.88*** | **1.99*** | 1.31 |
| *T2 vs. T1* | **1.48*** | **1.62*** | **1.70*** | **1.54*** | **1.56*** |
| *PR+ vs. PR-* | 0.94 | 0.91 | 0.98 | 0.97 | 0.93 |
| *Other race vs. White* | **0.77*** | **0.74*** | **0.75*** | **0.80*** | **0.67*** |
| *Black vs. White* | **1.33*** | **1.31*** | **1.31*** | **1.44*** | **1.27*** |
| *Neoadjuvant vs Adjuvant RT* | 1.00 | 1.00 | 1.01 | 1.01 | 1.00 |

* p-value < 0.05
